# Evaluation of contaminated drinking water and male breast cancer at Marine Corps Base Camp Lejeune, North Carolina: a case control study

**DOI:** 10.1186/s12940-015-0061-4

**Published:** 2015-09-16

**Authors:** Perri Zeitz Ruckart, Frank J. Bove, Edwin Shanley, Morris Maslia

**Affiliations:** Agency for Toxic Substances and Disease Registry, Division of Toxicology and Human Health Sciences, Atlanta, USA; National Center for Environmental Health/Agency for Toxic Substances and Disease Registry, Office of the Director, Atlanta, USA; Agency for Toxic Substances and Disease Registry, Division of Community Health Investigations, 4770 Buford Highway, MS F-58, Atlanta, GA 30341 USA

**Keywords:** Male breast cancer, Volatile organic compounds, Solvents, Drinking water, Military exposures

## Abstract

**Background:**

Solvents contaminated drinking water supplies at Marine Corps Base Camp Lejeune during 1950s-1985.

**Methods:**

We conducted a case–control study among Marines to evaluate associations between residential exposure to contaminated drinking water at Camp Lejeune and male breast cancer risk. The study included 71 male breast cancer cases and 373 controls identified from the Department of Veteran’s Affairs (VA) cancer registry whose military personnel records were available. Controls were selected from cancers not known to be associated with solvent exposure and included 270 skin cancers, 71 mesotheliomas, and 32 bone cancers. Base assignment and risk factor information came from military personnel and VA records. Groundwater contaminant fate/transport and distribution system models provided monthly estimated residential contaminant levels. We conducted exact logistic regression using the 50^th^ percentile level among exposed controls to create low and high exposure categories. We calculated 95 % confidence intervals (CIs) to indicate precision of effect estimates. Exploratory analyses used proportional hazards methods to evaluate associations between exposures and age at diagnosis.

**Results:**

After adjusting for age at diagnosis, race, and service in Vietnam, the odds ratio (OR) for ever stationed at Camp Lejeune was 1.14 (95 % CI: 0.65, 1.97). Adjusted ORs for high residential cumulative exposures to tetrachloroethylene (PCE), t-1,2 dichloroethylene (DCE), and vinyl chloride were 1.20 [95 % CI: 0.16-5.89], 1.50 [95 % CI: 0.30-6.11], 1.19 [95 % CI: 0.16-5.89], respectively, with a monotonic exposure response relationship for PCE only. However these results were based on two or three cases in the high cumulative exposure categories. Ever stationed at Camp Lejeune and high cumulative exposures to trichloroethylene (TCE), PCE, DCE and vinyl chloride were associated with earlier age at onset for male breast cancer; hazard ratios ranged from 1.4-2.7 with wide confidence intervals for cumulative exposure variables.

**Conclusion:**

Findings suggested possible associations between male breast cancer and being stationed at Camp Lejeune and cumulative exposure to PCE, DCE, and vinyl chloride. TCE, PCE, DCE and vinyl chloride cumulative exposures showed possible associations with earlier age at onset of male breast cancer. However, this study was limited by small numbers of cases in high exposure categories.

**Electronic supplementary material:**

The online version of this article (doi:10.1186/s12940-015-0061-4) contains supplementary material, which is available to authorized users.

## Introduction

In 1982, the United States Marine Corps (USMC) Base Camp Lejeune, North Carolina, was found to have drinking water supplies contaminated with specific volatile organic compounds including trichloroethylene (TCE), tetrachloroethylene (PCE), vinyl chloride, and benzene. The contamination began in the 1950s and continued until the most contaminated wells were removed from service in February 1985 [1, 2]. Details about the drinking water contamination have been published elsewhere [1–4]. The present study was prompted by concerns from the affected population that the drinking water exposures at Camp Lejeune may have caused male breast cancer.

Male breast cancer is a rare disease. The age-adjusted incidence rates for female and male breast cancer from CDC WONDER for the United States for 2011 (the most recent year data are available) were 122 per 100,000 and 1.4 per 100,000, respectively [5]. Several studies have examined the relationship between breast cancer and occupational exposure to solvents. Point estimates above 1 were found in several studies that evaluated female breast cancer and occupational exposure to solvents [6–13]. For male breast cancer, one study investigated occupational exposure to a wide-range of contaminants including industrial solvents and another study investigated exposure to gasoline and combustion by-products. The study on exposure to gasoline and combustion by-products found an OR of 2.5 (95 % CI: 1.3-4.5) for men with > 3 months employment in these industries and a lag time of at least 10 years; the OR was 5.4 (95 % CI: 2.4-11.9) among men who were under 40 years at the time of first employment in these industries [14]. Painters had an adjusted OR of 2.3 (95 % CI: 1.0-5.2) for male breast cancer while men employed as motor vehicle mechanics had an adjusted OR of 2.1 (95 % CI: 1.0-4.4) [15].

There are no studies which focused solely on associations between the contaminants found in the drinking water at Camp Lejeune and male breast cancer. One study conducted in Cape Cod, MA suggested an exposure response association between exposure to PCE contaminated drinking water and female breast cancer (adjusted ORs = 1.5, 95 % CI: 0.5-4.7 and 2.3, 95 % CI: 0.6-8.8 for the 75th percentile and 7 and 9 years of latency, respectively) [16, 17]. A recently published mortality study at Camp Lejeune could not evaluate male breast cancer because of small numbers of deaths in the Camp Lejeune cohort whose underlying cause was male breast cancer [4]. The purpose of this study was to evaluate whether residential exposure to the contaminated drinking water at Camp Lejeune increased the risk of male breast cancer incidence, using cases ascertained through the Department of Veterans Affairs Central Cancer Registry (VACCR).

## Methods

We used a case control study design to evaluate whether residential drinking water exposures at Camp Lejeune were associated with an increased risk of male breast cancer among Marines. Cases and controls were selected from among Marines included in the Department of Veteran’s Affairs Central Cancer Registry (VACCR). VACCR maintains information from eligible veterans who were diagnosed with or treated for cancer at a Department of Veteran’s Affairs (VA) clinic. VACCR provided the best currently available representative sample of cancer information for Marine veterans given the VA medical care system consists of 150 hospitals and over 800 clinics geographically dispersed around the US and US territories [18]. However, most of the nation’s veterans including most Marines do not use or are not eligible for VA care. Generally, only those with service-connected disabilities or who are low-income receive care from the VA [19]. For example, a 2010 National Survey of Veterans found that about 28 % of veterans used some form of VA care [20]. Informed consent was not obtained from participants because this was a data-linkage study that did not involve contact with participants.

### Study population

Eligible study members were male Marines born before January 1, 1969 and diagnosed with or treated for cancer at a VA medical facility from January 1, 1995, (the start of VACCR) to May 5, 2013 (the date for which complete VACCR data were available when the study was conducted) for whom we could identify tour dates and locations. We excluded those born after January 1, 1969, as these individuals were not old enough to serve during the period of contamination at Camp Lejeune (i.e. those at least 17 years of age by the end of 1985).

VACCR initially identified 78 incident cases of male breast cancer based on primary diagnosis and histological confirmation. To minimize the possibility of selection biases and ensure that the controls were representative of the source population, controls were selected from among incident cancers in the VACCR that are not known to be associated in the literature with solvent exposure [21]. Controls included non-melanoma skin cancers, bone cancers, and mesothelioma cancers of the pleura and peritoneum. VACCR data included 663 potential controls: 555 skin cancers, 32 bone cancers, 72 mesothelioma cancers of the pleura, and 4 mesothelioma cancers of the peritoneum. Because we planned to have 5 controls per case in the final sample, we included all cancers of the bone and mesothelioma and a random sample of 292 skin cancers for a total of 400 controls in the final sample. This study was approved by the Centers for Disease Control and Prevention (CDC) Institutional Review Board.

### Data collection

For each case and control, we obtained data from the National Personnel Record Center (NPRC) military personnel files to identify those who were stationed at Camp Lejeune before 1986. NPRC was able to locate 444 (92.8 %) of the 478 requested personnel files; files were unavailable for 7 (9.0 %) cases and 27 (6.8 %) controls (22 skin cancers and 5 cancers of the pleura). For each available personnel file, an extensive review and data abstraction process was conducted. Additional file 1 summarizes the data elements abstracted from the military personnel records. For tours when the Marine was stationed at Camp Lejeune, we used NPRC information to determine arrival and departure dates, unit, and marital status for each tour.

Information on potential risk factors was obtained from NPRC and two VA health records databases: VACCR and the VA’s Patient Treatment File (PTF); variables requested from VACCR and the PTF are listed in Additional file 2. The PTF captures data for in-patient services provided at a VA facility or that are paid for by the VA. VACCR and PTF records contained pertinent medical and demographic data, such as tumor characteristics (e.g. histological confirmation), socio-demographic information (e.g. date of birth, age at diagnosis, race, etc.), and medical conditions (e.g. diabetes, obesity, gynecomastia, and Klinefelter syndrome) that are or may be associated with male breast cancer [22–25]. PTF data on alcoholism, cholelithiasis, diabetes, diseases of the male genital organs, endocrine/metabolic/immune disease, fractures, gynecomastia, liver disease, obesity, orchitis/epididymitis, osteoporosis, and thyroid disorder were missing for 50 (13.4 %) controls and 5 (7.0 %) cases. Information on service in Vietnam, rank, and Military Occupational Specialty (MOS) codes came from NPRC records. A study in Europe found elevated rates of male breast cancer in men who were occupationally exposed to polychlorinated biphenyls and dioxin at levels at or above the median; dioxin is a by-product of Agent Orange [15]. For MOS codes, we evaluated possible exposures to solvents and electromagnetic fields (EMFs). Possible exposure to EMFs may be associated with male breast cancer [26].

### Exposure assessment

**Fig. 1:**
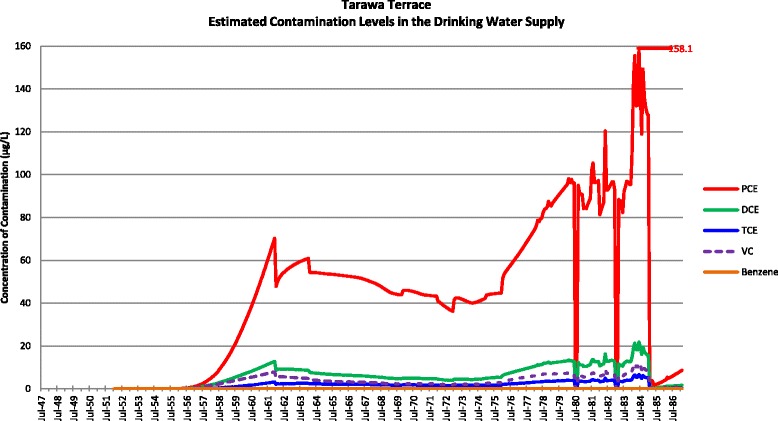
Estimated contamination levels in the drinking water supply for Tarawa Terrace

**Fig. 2:**
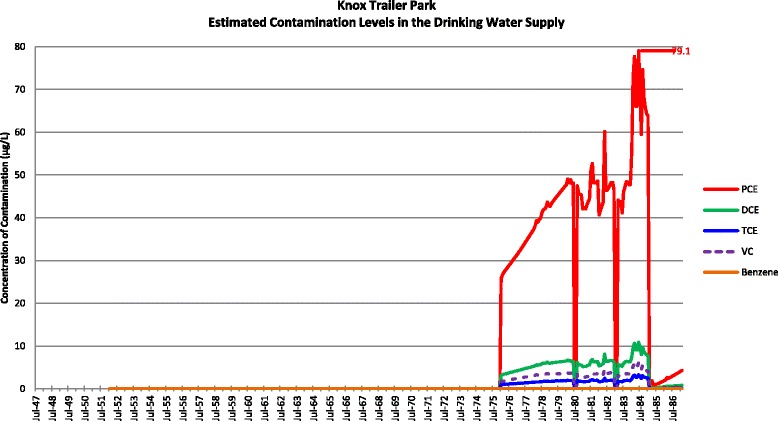
Estimated contamination levels in the drinking water supply for Knox Trailer Park. Note 1: Knox Trailer Park was constructed in 1976 [1], and analysis assumes that 1/2 of the drinking water supply was from the Tarawa Terrace distribution system

**Fig. 3:**
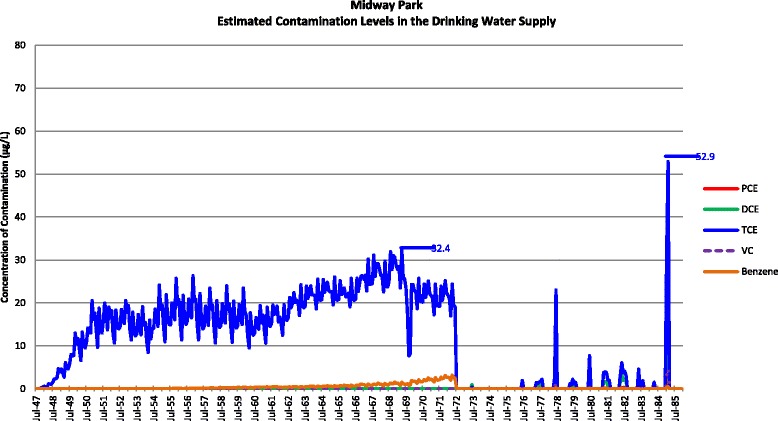
Estimated contamination levels in the drinking water supply for Midway Park

**Fig. 4:**
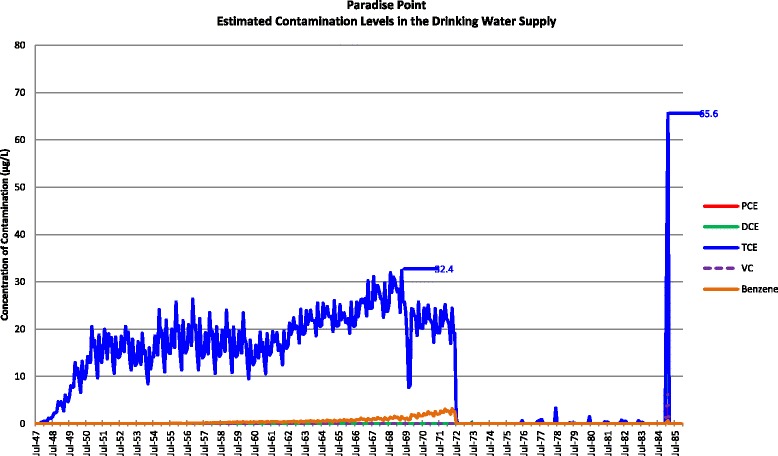
Estimated contamination levels in the drinking water supply for Paradise Point

**Fig. 5:**
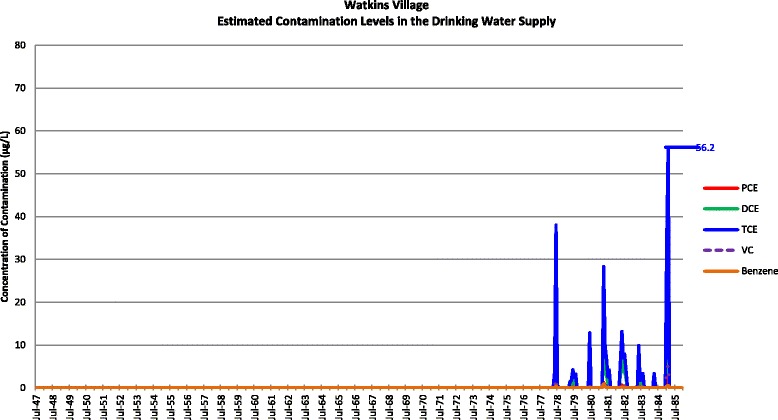
Estimated contamination levels in the drinking water supply for Watkins Village

**Fig. 6:**
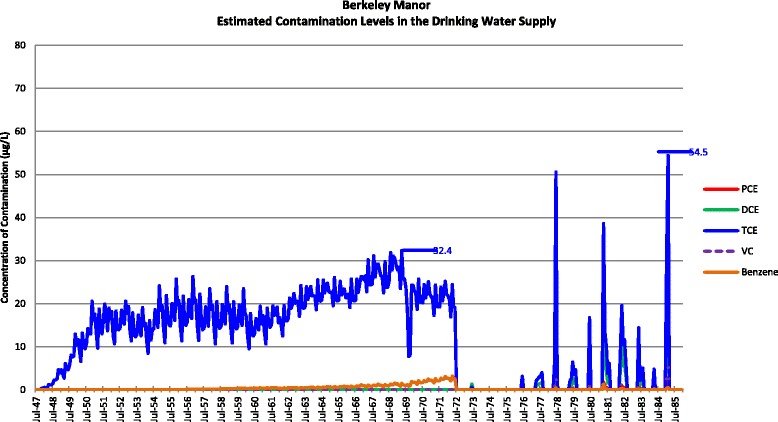
Estimated contamination levels in the drinking water supply for Berkeley Manor

**Fig. 7:**
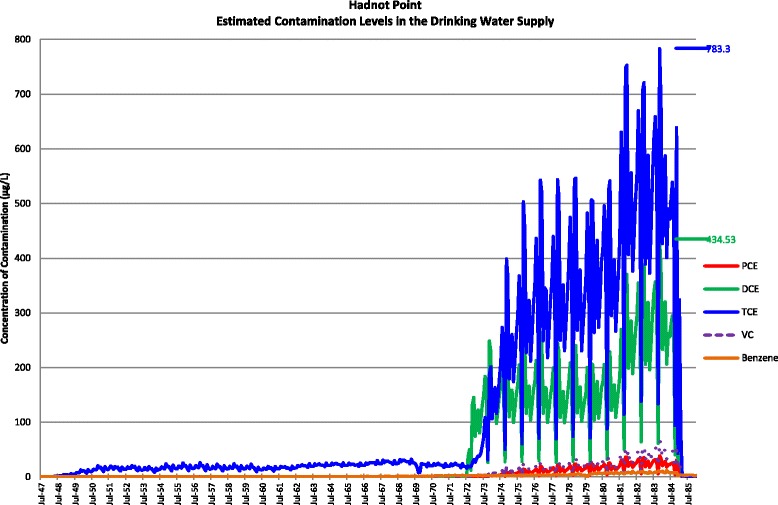
Estimated contamination levels in the drinking water supply for Hadnot Point

Actual contamination levels during most of the study period are unknown. The Agency for Toxic Substances and Disease Registry (ATSDR) conducted a historical reconstruction of the contamination levels in the Tarawa Terrace (TT), Hadnot Point (HP), and Holcomb Boulevard water treatment plants (WTPs) because only a few samples were taken in the distribution systems during 1980–1985 and there was a lack of contaminant-specific data. ATSDR’s historical reconstruction provided estimated monthly average contamination levels in drinking water delivered to residences (including barracks) served by the TT, HP and HB WTPs [see Figs. 1, 2, 3, 4, 5, 6 and 7]. The HP system served most of the barracks at the base from the start of operation through the end of the study period. The HP system also served a large number of family housing units and bachelors officers’ quarters until 1972 when the HB system began operation and served these housing areas. After June 1972, the family housing units served by the HP system included only those associated with the base hospital (“Hospital Point”). Family housing units were also served by the TT system. Detailed methodology and analysis of the water modeling activities were published in peer reviewed reports [1, 2]. We combined the water modeling results with information abstracted from NPRC, base family housing records, and information on where units were barracked to assign contaminant-specific residential exposure levels to each case and control who were stationed at Camp Lejeune.

To determine residence, we assumed that: (1) unmarried enlisted Marines resided in barracks; (2) unmarried officers resided in the bachelors officers’ quarters (BOQs) in the area where their units were barracked; and (3) married Marines resided either in off-base housing or in base family housing. Unit barrack locations were identified using information provided by retired Marines, base staff, and base command chronologies. Married Marines usually resided either in off-base housing or in base family housing; however when names of married Marines were not found in family housing records and their spouse’s address was not in or near the Jacksonville area, we assumed they were barracked with their unit.

The exposure period was the earliest start date of a tour at Camp Lejeune and continued until the Marine left Camp Lejeune or December 31, 1985, whichever was earlier. When determining exposure, we took into account Marines who had more than one tour at Camp Lejeune and who may have left the base and come back. All tours not at Camp Lejeune were assigned as unexposed. For each tour at Camp Lejeune, a Marine was categorized as unexposed during that tour if residing off-base or at a residence at Camp Lejeune that received uncontaminated drinking water. To assign categories of exposure, we used the estimated average monthly contaminant concentrations in the drinking water system serving the individual’s residence(s) at Camp Lejeune to determine average and cumulative exposure to the contaminants (parts per billion [ppb]-months). Monthly estimates were weighted by the proportion of days per month that the Marine lived in a residence that received contaminated drinking water. For those with any exposure to a specific contaminant, the average exposure was equal to the cumulative exposure from that contaminant divided by the months the individual had an exposure to that contaminant (i.e., the number of months that the drinking water serving the individual’s residence had levels of the contaminant > 0).

### Data analysis

We used exact logistic regression and conditional logistic regression in SAS 9.3 to compare the odds of male breast cancer among the exposure variables [27]. Exact and conditional logistic regressions are used when data are sparse [21, 28]. In order to conduct the conditional logistic regression models, we introduced matching on age at diagnosis. Unadjusted and adjusted odds ratios (ORs and aORs), their 95 % confidence intervals (CIs), and p-values were calculated. For the exact logistic regression, we calculated the mid-p CI and p-value. Potential confounding by the risk factors listed in Table 1 was determined by comparing the unadjusted model for the exposure variable that included only those with complete data on the risk factor with the model adjusted for the risk factor using a 10 % change in the estimate rule [29]. All adjusted models included age at diagnosis and race because of their known association with male breast cancer. Because diabetes and gynecomastia were the only variables from the PTF that changed ORs by >10 % in the initial screening, we evaluated these two risk factors by conducting multiple imputation using SAS 9.3 PROC MI and PROC MIANALYZE to impute values for these variables with missing data. The monotone missing pattern method was specified and logistic regression was used with 40 iterations. Predictor variables included case/control status, age at diagnosis (continuous variable), whether the individual served in Vietnam, race, ethnicity, rank, and the exposure variable under evaluation. The remaining selected potential confounders without missing data were included singly in an age- and race-adjusted model. A final model was chosen based on which variables (including imputed values of diabetes and gynecomastia) caused > 10 % change in the estimate that only included age at diagnosis and race.

**Table 1:** Characteristics of the cases and controls

Characteristic	Controls		Cases		OR (95 % CI)
	#	%	#	%	
Age at diagnosis, in years^a^					
<50	31	8.3	5	7.0	1.00 (ref.)
≥50 - <60	72	19.3	22	31.0	1.89 (0.68-6.05)
≥60 - <80	241	64.6	40	56.3	1.03 (0.40-3.14)
≥80	29	7.8	4	5.7	0.86 (0.19-3.71)
Ethnicity^a^					
Not Hispanic	362	97.1	67	94.4	1.00 (ref.)
Hispanic	11	2.9	4	5.6	1.96 (0.53-6.16)
Race^a^					
White	345	92.7	55	78.6	1.00 (ref.)
Black/other^b^	27	7.3	15	21.4	3.49 (1.74-6.96)
Rank^c^					
Officer	9	2.4	1	1.4	1.00 (ref.)
Enlisted	364	97.6	70	98.6	1.73 (0.28-38.78)
Served in Vietnam^c^					
No	288	77.2	40	56.3	1.00 (ref.)
Yes	85	22.8	31	43.7	2.62 (1.54-4.45)
Alcoholism^d^					
No	251	77.7	49	74.2	1.00 (ref.)
Yes	72	22.3	17	25.8	1.21 (0.64-2.21)
Thyroid disorder^d^					
No	295	91.3	63	95.5	1.00 (ref.)
Yes	28	8.7	3	4.5	0.50 (0.12-1.55)
Endocrine/metabolic/immune disease^d^					
No	90	27.9	8	12.1	1.00 (ref.)
Yes	233	72.1	58	87.9	2.79 (1.33-6.49)
Diabetes^d^					
No	246	76.2	36	54.5	1.00 (ref.)
Yes	77	23.8	30	45.5	2.66 (1.53-4.60)
Obesity^d^					
No	299	92.6	56	84.9	1.00 (ref.)
Yes	24	7.4	10	15.2	2.22 (0.97-4.84)
Cholelithiasis^d^					
No	301	93.2	64	97.0	1.00 (ref.)
Yes	22	6.8	2	3.0	0.43 (0.07-1.62)
Diseases of the male genital organs^d^					
No	235	72.8	48	72.7	1.00 (ref.)
Yes	88	27.2	18	27.3	1.00 (0.54-1.80)
Orchitis/epididymitis^d^					
No	319	98.8	65	98.5	1.00 (ref.)
Yes	4	1.2	1	1.5	1.23 (0.05-9.94)
Gynecomastia^d^					
No	321	99.4	57	86.4	1.00 (ref.)
Yes	2	0.6	9	13.6	25.01 (5.78-173.64)
Osteoporosis^d^					
No	293	90.7	59	89.4	1.00 (ref.)
Yes	30	9.3	7	10.6	1.16 (0.45-2.68)
Fractures^d^					
No	302	93.5	57	86.4	1.00 (ref.)
Yes	21	6.5	9	13.6	2.27 (0.94-5.14)
Liver disease^d^					
No	296	91.6	59	89.4	1.00 (ref.)
Yes	27	8.4	7	10.6	1.30 (0.55-3.04)
Non-alcoholic liver disease^d^					
No	313	96.9	63	95.5	1.00 (ref.)
Yes	10	3.1	3	4.5	1.49 (0.32-5.30)
MOS^e^, possible EMF^f^ exposure^c^					
No	368	98.7	68	95.8	1.00 (ref.)
Yes	5	1.3	3	4.2	3.25 (0.76-13.91)
MOS^e^, possible solvent exposure^c^					
No	274	73.5	50	70.4	1.00 (ref.)
Yes	99	26.5	21	29.6	1.16 (0.67-2.03)
Birth cohort^a^					
≤1925	90	24.1	8	11.3	1.00 (ref.)
1926-1935	121	32.4	16	22.5	1.49 (0.61-3.63)
1936-1945	66	17.7	14	19.7	2.39 (0.95-6.02)
1946-1950	60	16.1	21	29.6	3.94 (1.64-9.47)
≥1950	36	9.7	12	16.9	3.75 (1.42-9.94)

^a^obtained from VACCR; ^b^includes 2 controls who were “other” race; ^c^obtained from NPRC; ^d^obtained from PTF; ^e^Military Occupational Specialty; ^f^Electromagnetic fields

We used two criteria to assess associations: magnitude of the OR and the exposure-response relationship. If an exposure-response relationship could be evaluated, emphasis was given to monotonic trends in the categorical exposure variables. A monotonic trend occurs when every change in the OR with increasing category of exposure is in the same direction, although the trend could have flat segments but never reverse direction [21]. Confidence intervals were used to indicate precision of ORs. We included p-values in tables for information purposes only. We did not use statistical significance testing to interpret findings [21, 30, 31]. We emphasize the point estimate as a measure of the effect of exposure, use the confidence interval as an indicator of the level of uncertainty or precision of the point estimate, and consider the possible sources of bias [32].

Cumulative exposure (ppb-months) to each contaminant was evaluated separately. We also evaluated the sum of the amount of all the contaminants (“TVOC”), the sum of the amount of the chlorinated contaminants (PCE, TCE, DCE, and vinyl chloride), and the sum of the amount of the known or probable carcinogenic contaminants (PCE, TCE, benzene, and vinyl chloride). Exposure variables were categorized such that the reference group did not have residential exposure to the contaminant under evaluation (“unexposed”). For the categorical analyses of cumulative exposure, we divided the exposed group into low and high exposure categories using the 50^th^ percentile level among exposed controls. To evaluate exposure-response trends, continuous (untransformed and log base 10 transformed) exposure variables for cumulative exposure and average exposure were evaluated in logistic regressions and restricted cubic splines with three knots (with and without their 95 % confidence intervals) were produced [33]. Exposure-response trends were also evaluated in logistic regression models using the median value for each categorical exposure level.

We also evaluated average monthly exposure in a manner similar to that of cumulative exposure. Additionally, we compared the duration of Marines residing in areas served by contaminated drinking water at Camp Lejeune to Marines who were never stationed at Camp Lejeune or who resided in areas at Camp Lejeune that did receive contaminated drinking water. Duration was classified into two categories using the median number of weeks residing in areas served by contaminated drinking water. Finally, we compared those stationed at any time at Camp Lejeune with those who were never stationed at Camp Lejeune because Marines at Camp Lejeune who resided in areas that did not receive contaminated drinking water may have been exposed to the contaminated drinking water during training exercises or other activities on base.

Exploratory analyses using proportional hazards methods were used to evaluate whether being stationed at Camp Lejeune and cumulative exposures to the drinking water contaminants were associated with earlier age at onset for male breast cancer. Age at diagnosis as a continuous variable was the response variable in the proportional hazards model (i.e., age was the “time variable”). These analyses used methods developed for case-cohort samples that assume the controls in this study approximate a hypothetical sample of the underlying “cohort” that gave rise to the cases. Such an assumption would hold if the male breast cancer rate in the underlying population is low [34]. The lifetime risk of being diagnosed with male breast cancer is about 1 in 1000 in the United States [35]. The incidence rate for men 50 years of age and older is about 4 per 100,000 person-years according to data from the Surveillance, Epidemiology, and End Results (SEER) Program of the National Cancer Institute. We used four proportional hazards methods to model age at diagnosis [36–38]. The four methods varied in how they weighted the cases and non-cases in order to account for oversampling of cases. Cases were oversampled because, in the case-cohort approach, a sample is taken of the entire cohort (“the cohort subsample”), but all the cases in the entire cohort are also included. The four methods also varied in whether they used an exact or approximate pseudolikelihood to estimate the regression coefficients and their variances. Models were adjusted for race and service in Vietnam. Tied failure times (ages) were randomly broken by assigning slightly different ages at diagnosis (e.g., age + 0.3 years) so that each case had a unique age at diagnosis [36]. Each of the four methods produced similar hazard ratios (HRs) for the categorical exposure variables. A robust or sandwich variance estimator was used to compute 95 % confidence intervals [36].

## Results

A total of 71 cases of male breast cancer and 373 controls that had personnel records at NPRC were included in the study. Of the 373 controls, 270 (72.3 %) were skin cancers, 67 (18.0 %) were mesotheliomas of the pleura, 32 (8.6 %) were bone cancers, and 4 (1.1 %) were mesotheliomas of the peritoneum. One control had enough information in the personnel file to determine that he was stationed at Camp Lejeune, but there was no information on the individual’s unit or dates of tour arrival and departure. We retained this control in analyses comparing those stationed at Camp Lejeune with those never stationed at Camp Lejeune; however, we excluded this control from analysis evaluating contaminant-specific levels and duration of exposure.

Potential risk factors from VACCR, NPRC, and the PTF are shown in Table 1. Race was missing for one case and one control; we classified these participants as “white”. However, as a check we compared the ORs for the exposure variables when both participants were classified first as “white” and then as “other race” but observed no differences. We could not evaluate Klinefelter syndrome because there were no study subjects with PTF information that had this condition. MOS was categorized into two dichotomous variables: any occupation with potential solvent exposure and any occupation with potential EMF exposure. Available VACCR data on characteristics of the 34 Marines (7 [9.0 %] cases and 27 [6.8 %] controls) whose records were not located at NPRC are provided in Table 2. Race distribution was similar between the Marines used in the analyses and the Marines whose personnel records could not be located; however there were no Hispanics among those whose records could not be located. Most cases with and without records at NPRC were diagnosed when they were 60 years of age or older. Marines whose records could not be located were slightly older than Marines used in the analyses. Regardless of whether NPRC records were available, the majority of the cases were diagnosed after 2001 whereas a majority of the controls were diagnosed prior to 2002. Over the last 10–15 years, the annual age adjusted incidence rate for male breast cancer in the United States has been increasing slightly according to data from CDC WONDER [5]. Our data indicated that the incidence rate for male breast cancer in the VACCR was increasing much faster than the national rate, at least among Marines. In our study, slightly over 60 % of the male breast cancer cases occurred during 2004–2012 while about 32 % of the control cancers occurred during this period. The more recently diagnosed male breast cancer cases tended to be from Marines who were not stationed at Camp Lejeune. For example, during the period 2004–2012, 68 % of the non-Camp Lejeune cases and 50 % of the Camp Lejeune cases were diagnosed.

**Table 2:** Comparison of non-participating cases and controls on selected characteristics^*^

Characteristic	Controls (*n* = 27)		Cases (*n* = 7)	
	#	%	#	%
Age at diagnosis, in years				
<50	4	14.8	0	0
≥50 - <60	2	7.4	1	14.3
≥60 - <80	19	70.4	6	85.7
≥80	2	7.4	0	0
Race				
White	24	88.9	5	71.4
Black/other	2	7.4	2	28.6
Birth cohort				
≤1925	9	33.3	0	0
1926-1935	10	37.0	4	57.1
1936-1945	1	3.7	2	28.6
1946-1950	4	14.8	0	0
≥1950	3	11.1	1	14.3

^a^data available from VACCR

Results from the conditional and exact logistic regressions were similar so therefore, we decided to present exact logistic regression results. When Marines stationed at Camp Lejeune were compared with those never stationed at Camp Lejeune, the unadjusted OR for male breast cancer was 1.45 (95 % CI: 0.86-2.44) and the aOR was 1.14 (95 % CI: 0.65-1.98), adjusted for age at diagnosis, race, and service in Vietnam (Table 3). The aOR was 0.89 (95 % CI: 0.38-1.93) for duration ≥ 38 weeks in a residence receiving contaminated drinking water at Camp Lejeune.

**Table 3:** Crude and adjusted ORs for male breast cancer by exposure status

Exposure	Controls	Cases	OR (95 % CI)	*p*-value	aOR^a^ (95 % CI)	*p*-value
	# (%)	# (%)				
Ever stationed at Camp Lejeune						
No	248 (66.5)	41 (57.7)	1.00 (ref.)		1.00 (ref.)	
Yes	125 (33.5)	30 (42.3)	1.45 (0.86-2.44)	0.16	1.14 (0.65-1.97)	0.65
Cumulative PCE						
No exposure	357 (96.0)	67 (94.4)	1.00 (ref.)		1.00 (ref.)	
Low (>0- < 36 ppb-months)	7 (1.9)	2 (2.8)	1.52 (0.21-7.00)	0.59	1.05 (0.14-5.14)	0.91
High (≥36 ppb-months)	8 (2.2)	2 (2.8)	1.33 (0.19-5.91)	0.69	1.20 (0.16-5.89)	0.80
Cumulative TCE						
No exposure	258 (69.4)	46 (64.8)	1.00 (ref.)		1.00 (ref.)	
Low (>0- <159 ppb-months)	57 (15.3)	13 (18.3)	1.28 (0.63-2.49)	0.48	1.06 (0.50-2.13)	0.86
High (≥159 ppb-months)	57 (15.3)	12 (16.9)	1.18 (0.57-2.34)	0.63	0.93 (0.43-1.90)	0.85
Cumulative benzene						
No exposure	260 (69.9)	46 (64.8)	1.00 (ref.)		1.00 (ref.)	
Low (>0- < 3.6 ppb-months)	57 (15.3)	15 (21.1)	1.49 (0.76-2.82)	0.24	1.67 (0.82-3.30)	0.15
High (≥3.6 ppb-months)	55 (14.8)	10 (14.1)	1.03 (0.47-2.18)	0.92	0.57 (0.24-1.25)	0.17
Cumulative DCE						
No exposure	356 (95.7)	67 (94.4)	1.00 (ref.)		1.00 (ref.)	
Low (>0- <472 ppb-months)	8 (2.2)	1 (1.4)	0.67 (0.03-4.25)	0.78	0.56 (0.02-3.83)	0.67
High (≥472 ppb-months)	8 (2.2)	3 (4.2)	1.99 (0.42-7.47)	0.34	1.50 (0.30-6.11)	0.57
Cumulative vinyl chloride						
No exposure	356 (95.7)	67 (94.4)	1.00 (ref.)		1.00 (ref.)	
Low (>0- <60 ppb-months)	8 (2.2)	2 (2.8)	1.33 (0.19-5.89)	0.69	0.94 (0.13-4.38)	0.99
High (≥60 ppb-months)	8 (2.2)	2 (2.8)	1.33 (0.19-5.89)	0.69	1.19 (0.16-5.89)	0.81
Cumulative TVOC^b^						
No exposure	258 (84.9)	46 (64.8)	1.00 (ref.)		1.00 (ref.)	
Low (>0- <168 ppb-months)	57 (15.3)	14 (19.7)	1.38 (0.69-2.64)	0.35	1.18 (0.57-2.33)	0.64
High (≥168 ppb-months)	57 (15.3)	11 (15.5)	1.08 (0.51-2.18)	0.81	0.82 (0.37-1.72)	0.63
Monthly average PCE						
No exposure	357 (96.0)	67 (94.4)	1.00 (ref.)		1.00 (ref.)	
Low (>0- < 2 ppb)	8 (2.2)	2 (2.8)	1.33 (0.19-5.90)	0.69	0.91 (0.13-4.21)	0.96
High (≥2 ppb)	7 (1.9)	2 (2.8)	1.52 (0.21-7.00)	0.59	1.47 (0.18-7.91)	0.65
Monthly average TCE						
No exposure	258 (69.4)	46 (64.8)	1.00 (ref.)		1.00 (ref.)	
Low (>0- <17.5 ppb)	57 (15.3)	8 (11.3)	0.79 (0.33-1.71)	0.58	1.02 (0.42-2.30)	0.93
High (≥17.5 ppb)	57 (15.3)	17 (23.9)	1.67 (0.88-3.10)	0.12	0.97 (0.47-1.94)	0.94
Monthly average benzene						
No exposure	260 (69.9)	46 (64.8)	1.00 (ref.)		1.00 (ref.)	
Low (>0- < 0.4 ppb)	56 (15.1)	9 (12.7)	0.91 (0.40-1.92)	0.83	1.23 (0.52-2.72)	0.61
High (≥0.4 ppb)	56 (15.1)	16 (22.5)	1.61 (0.83-3.03)	0.15	0.87 (0.42-1.78)	0.72
Monthly average DCE						
No exposure	356 (95.7)	67 (94.4)	1.00 (ref.)		1.00 (ref.)	
Low (>0- < 94 ppb)	8 (2.2)	2 (2.8)	1.33 (0.20-5.90)	0.69	0.88 (0.12-4.01)	0.92
High (≥94 ppb)	8 (2.2)	2 (2.8)	1.33 (0.20-5.90)	0.69	1.32 (0.17-6.77)	0.73
Monthly average vinyl chloride						
No exposure	356 (95.7)	67 (94.4)	1.00 (ref.)		1.00 (ref.)	
Low (>0- <3.4 ppb)	8 (2.2)	2 (2.8)	1.33 (0.20-5.90)	0.69	0.94 (0.13-4.37)	0.99
High (≥3.4 ppb)	8 (2.2)	2 (2.8)	1.33 (0.20-5.90)	0.69	1.19 (0.16-5.89)	0.81
Monthly average TVOC^b^						
No exposure	258 (69.4)	46 (64.8)	1.00 (ref.)		1.00 (ref.)	
Low (>0- <18 ppb)	58 (15.6)	9 (12.7)	0.87 (0.38-1.83)	0.75	1.15 (0.49-2.51)	0.72
High (≥18 ppb)	56 (15.1)	16 (22.5)	1.60 (0.83-3.01)	0.16	0.89 (0.43-1.80)	0.76

^a^adjusted for age at diagnosis, race, and service in Vietnam; ^b^sum of the amount of exposure to PCE, TCE, benzene, DCE, and vinyl chloride

We focused on results for cumulative exposure because they were generally similar to results using average monthly exposure. However, we highlighted results of average exposure when they differed markedly from cumulative exposure. We also presented TVOC results and not the sum of the amount of chlorinated or known/probable carcinogenic contaminants because both were similar to results obtained for TVOC. For the evaluation of exposure-response trends, results of analyses using the medians of the categorical exposure levels in the logistic regression models were similar to analyses using the untransformed cumulative exposure variables. Moreover, the untransformed cumulative exposure variables had lower AIC values than the log-transformed variables, so only the results for untransformed variables are presented.

Adjusted ORs for cumulative exposure among those with high exposure were 1.20 (95 % CI: 0.16-5.89) for PCE, 1.50 (95 % CI: 0.30-6.11) for DCE, and 1.19 (95 % CI: 0.16-5.89) for vinyl chloride, and there was a monotonic exposure response relationship for PCE (Table 3). The aORs for PCE and vinyl chloride were based on two cases with high exposure and the aOR for DCE was based on three cases with high exposure. The unadjusted ORs for cumulative exposure to these contaminants were higher than the results adjusted for age at diagnosis, race, and service in Vietnam. The logistic regression parameter estimates for the continuous cumulative exposure variables for PCE, DCE and vinyl chloride were 0.012 ppb-years (standard error [SE] = 0.069, *p* = 0.77), 0.003 ppb-years (SE = 0.003, *p* = 0.35) and 0.021 ppb-years (SE = 0.044, *p* = 0.58), respectively.

For high cumulative exposure to TCE, benzene, and TVOC, unadjusted ORs were >1.00 (ORs were 1.18 [95 % CI: 0.57-2.34], 1.03 [95 % CI: 0.47-2.18], and 1.08 [95 % CI: 0.51-2.18], respectively), but adjusted results were not elevated. The parameter estimates for the continuous exposure variables for TCE, benzene and TVOC were 0.001 ppb-years (SE = 0.002, *p* = 0.56), −0.024 ppb-years (SE = 0.133, *p* = 0.86) and .001 ppb-years (SE= 0.001, *p* = 0.48), respectively. The splines for all the cumulative exposure variables were somewhat J-shaped indicating non-monotonic trends with ORs falling below 1.0 for low and medium exposures and rising above 1.0 at the higher exposure levels (see Additional file 3: Figures S8–S15).

For the adjusted analyses of average exposures to TCE, benzene, VC, and TVOC, results were similar to those for cumulative exposures. The adjusted ORs for high average exposure were greater for PCE (1.47, 95 % CI: 0.18-7.91) and lower for DCE (1.32, 95 % CI: 0.17-6.77) compared to aORs for high cumulative exposure to these chemicals. For average exposure to PCE, there was an exposure-response relationship for the unadjusted OR analysis; these results were based on two cases with low exposure and two cases with high exposure. Parameter estimates for the continuous average exposure variables were negative except for DCE (β = 0.001, SE = 0.006, *p* = 0.73), and only the spline for DCE indicated a monotonic exposure-response.

For those with PTF data, there appeared to be possible confounding by diabetes and gynecomastia when evaluated separately with each exposure variable. However, because of missing data, we used a multiple imputation procedure to impute values for the missing data for diabetes and gynecomastia. Including imputed values of diabetes and gynecomastia in a model that also included age at diagnosis, service in Vietnam, and race produced results similar to those obtained when diabetes and gynecomastia were removed from the model.

The results of the exploratory analyses of age at onset of male breast cancer are shown in Table 4. When Marines stationed at Camp Lejeune were compared with those never stationed at Camp Lejeune, the adjusted hazard ratio (HR) was 1.51 (95 % CI: 0.78, 2.95). Adjusted HRs for cumulative exposure among those with high exposure to PCE, TCE, DCE and vinyl chloride were 2.08 (95 % CI: 0.31, 14.00), 1.41 (95 % CI: 0.58, 3.46), 2.72 (95 % CI: 0.52, 14.18), and 2.14 (95 % CI: 0.31, 14.81), respectively. The parameter estimates for the continuous cumulative exposure variables for PCE, TCE, DCE, vinyl chloride and TVOC were 0.086 ppb-years (SE = 0.073, *p* = 0.23), 0.003 ppb-years (SE = 0.003, *p* = 0.36), 0.003 ppb-years (SE = 0.004, *p* = 0.40), 0.059 ppb-years (SE = 0.052, *p* = 0.25), and 0.001 ppb-years (SE = 0.002, *p* = 0.38), respectively. The parameter estimate for benzene was negative.

**Table 4:** Adjusted hazard ratios for age at onset of male breast cancer by exposure status (*n* = 444)

Exposure	Controls	Cases	Hazard Ratio	95 % Confidence Interval
Ever stationed at Camp Lejeune				
No	248	41	1.00 (ref.)	
Yes	125	30	1.51	0.78 - 2.95
Cumulative PCE				
No exposure	357	67	1.00 (ref.)	
Low (>0- < 36 ppb-months)	7	2	1.19	0.20 - 7.07
High (≥36 ppb-months)	8	2	2.08	0.31 - 14.00
Cumulative TCE				
No exposure	258	46	1.00 (ref.)	
Low (>0- <159 ppb-months)	57	13	1.13	0.49 - 2.62
High (≥159 ppb-months)	57	12	1.41	0.58 - 3.46
Cumulative benzene				
No exposure	260	46	1.00 (ref.)	
Low (>0- < 3.6 ppb-months)	57	15	1.95	0.93 - 4.10
High (≥3.6 ppb-months)	55	10	0.76	0.28 - 2.11
Cumulative DCE				
No exposure	356	67	1.00 (ref.)	
Low (>0- <472 ppb-months)	8	1	0.64	0.06 - 7.01
High (≥472 ppb-months)	8	3	2.72	0.52 - 14.18
Cumulative vinyl chloride				
No exposure	356	67	1.00 (ref.)	
Low (>0- <60 ppb-months)	8	2	1.17	0.20 - 6.89
High (≥60 ppb-months)	8	2	2.14	0.31 - 14.81
Cumulative TVOC^a^				
No exposure	258	46	1.00 (ref.)	
Low (>0- <168 ppb-months)	57	14	1.28	0.56 - 2.91
High (≥168 ppb-months)	57	11	1.21	0.48 - 3.04

^a^sum of the amount of exposure to PCE, TCE, benzene, DCE, and vinyl chloride

## Discussion

We observed ORs above 1.00 for ever being stationed at Camp Lejeune and for cumulative and average exposures to PCE, DCE, and vinyl chloride in the high exposure category. The ORs remained above 1.00 after adjusting for age at diagnosis, race, and service in Vietnam. However, these results had wide confidence intervals and were based on two or three cases with high exposure. A monotonic exposure-response relationship was observed only for categorized cumulative exposure to PCE based on two exposed cases in both the low and high exposure categories. For categorized cumulative and average exposures to DCE and vinyl chloride, as well as average exposure to PCE, the exposure-response relationships were not monotonic since the adjusted ORs at the low exposure level were below 1.00. With the exception of average exposure to DCE, none of the splines indicated a monotonic exposure-response relationship. No increased risk was found for duration of exposure.

The OR for the high categorical level of cumulative exposure to PCE, 1.20, is similar to ORs observed in the Cape Cod study for PCE and female breast cancer (aOR for women exposed above the median PCE level was 1.3 [95 % CI: 0.8-2.2] when 11 years of latency were considered and 1.2 [95 % CI: 0.6-2.4] when 15 years of latency were considered [17]. ORs in the Cape Cod study were increased at higher levels of PCE exposure (i.e., >75th percentile) and ranged from 1.5-1.9 for latencies up to 15 years. The latency period in the current study is ≥ 10 years because the most recent drinking water exposures occurred in February 1985 and VACCR began collecting data on January 1, 1995. The OR of 1.2 for the high categorical level of cumulative exposure to PCE is within the range of effect estimates observed in studies of occupational exposure to solvents and female breast cancer (SIRs ranged from 1.09-1.48 and SMRs ranged from 1.14-1.66) [7–9, 11, 12]. ORs in the current study are lower than those observed in a study of occupational exposure to benzene and female breast cancer (OR = 1.95, 95 % CI: 1.14-3.33 for high benzene exposure) [10] and male breast cancer (OR = 2.5, 95 % CI: 1.3-4.5) [14]. The ORs in the current study were also lower than those found in a study of male breast cancer and men employed as painters (aOR of 2.3, 95 % CI: 1.0-5.2) and men employed as motor vehicle mechanics (aOR of 2.1 (95 % CI: 1.0-4.4) [15].

The levels of at least one drinking water contaminant at Camp Lejeune were estimated to exceed current US EPA drinking water standards of 5 ppb for PCE, TCE, and benzene and 2 ppb for vinyl chloride during August 1953 and January 1985 [1, 2]. From January 1975 through February 1985 (when the highly contaminated wells were shut down), the average monthly level of TCE in the drinking water at HP was estimated at 359 ppb with an estimated range as high as 783 ppb; the average monthly level of vinyl chloride in the drinking water at HP was estimated at 24 ppb with an estimated range as high as 67 ppb, and the average monthly level of PCE in the drinking water at TT was estimated at 76 ppb with an estimated range as high as 158 ppb. Daily exposure to these levels of TCE in the drinking water (via ingestion, inhalation and dermal routes) is comparable to inhalation exposures that occur in some occupational settings [4]. Most of the levels of PCE in the Cape Cod study were within the range of 1–80 ppb except for areas of the piping where water was stationary for longer periods of time and there was more opportunity for PCE to leach from the pipe [17]. Camp Lejeune also had PCE levels in the 1–80 ppb range except for Tarawa Terrace after 1978 when the PCE levels were consistently above 80 ppb.

We conducted exploratory analyses using proportional hazards methods to evaluate whether being stationed at Camp Lejeune and cumulative exposures to the contaminants were associated with earlier age at onset for male breast cancer. This approach evaluates whether exposures accelerate the onset of a disease and therefore offers an alternative perspective than that provided by logistic regression analyses, which focuses solely on the presence or absence of the disease. Proportional hazards methods for the analysis of case–control age-at-onset data are appropriate if the disease of interest is rare in the source population [33]. We observed an accelerated onset of male breast cancer among those stationed at Camp Lejeune compared to other bases as well as among those exposed to higher cumulative levels of PCE, TCE, DCE and vinyl chloride. These results provide additional support to the associations observed in the logistic regression analyses for being stationed at Camp Lejeune and cumulative exposures to PCE, DCE and vinyl chloride. Cumulative exposure to TCE in the high exposure category was also observed to accelerate the onset of male breast cancer but was not observed to increase the risk of male breast cancer in the logistic regression analysis.

### Limitations

Several constraints within the available data are important for understanding the study’s limitations. Findings from this study were based on a small number of exposed male breast cancer cases resulting in wide confidence intervals for the estimated ORs. We were unable to include seven cases of male breast cancer in the analyses because we had no information about where they were stationed. Obtaining data for a larger proportion of the Marine population was not currently feasible for this study because it would have required obtaining data from a majority of state cancer registries. Only about 25 % of veterans reported using VA health care facilities [20]; therefore cases were likely underestimated which limited the sample size in this study. While missing cases who were diagnosed at non-VA facilities reduced the power of the study, this limitation was unlikely to have led to selection bias because it was unlikely that getting health care at the VA or elsewhere would be associated with exposure status during the timeframe when this study was conducted. Because some contaminants were correlated (e.g., PCE, DCE, and vinyl chloride) and we had small numbers of cases, it was difficult to separate effects of one chemical from another. Furthermore, we were unable to evaluate more than one chemical in a model because of small numbers of cases.

We did not conduct interviews to obtain more detailed information on residential history at Camp Lejeune or activities that may have resulted in drinking water exposures such as field training exercises at the base. Therefore, it is probable that exposure misclassification occurred which is likely to be non-differential because exposure information came from military personnel records and not from study members. For a small number of study participants, tour start and end dates at Camp Lejeune were inferred from additional information contained in personnel records because exact dates were not recorded. Additionally, exposure misclassification may have occurred as a result of assigning exposure levels based on residential location. Assigning exposure based solely on residence may not accurately represent the true level of drinking water exposure because the daily activities of each Marine (e.g., location and duration of field training) were unknown. It is likely that considerable amounts of drinking water were consumed during field training (via ingestion and showering) during hot summer months. Depending on training location, the water consumed could have been supplied by the HP system. However, it is likely that the exposure misclassification bias would be non-differential and would tend to bias results toward the null for dichotomous exposure variables. For categorical exposure variables with more than two levels or continuous exposure variables, non-differential exposure misclassification would tend to distort the exposure-response relationship, for example, resulting in underestimates or overestimates of the ORs at the lower exposure levels and an underestimate of the OR at the highest exposure level [21].

Although information from the PTF file was available on several medical conditions that are either known or possible risk factors for male breast cancer, PTF data were missing for 5 cases and 50 controls, requiring the use of a multiple imputation procedure. However, including diabetes and gynecomastia in the adjusted models after imputing values for those with missing data did not appreciably change the results for any of the analyses. The other medical conditions were not confounders when the analyses were restricted to those with PTF data. It is possible that confounding by unmeasured risk factors could have affected the findings in this study, resulting in underestimates or overestimates of the ORs. For example, we did not have information on BRCA1 and BRCA2 mutations, family history of breast cancer, and occupations before or after military service.

## Conclusion

ORs observed at the high level of the categorical cumulative exposure variables for PCE, DCE and vinyl chloride are suggestive of possible associations with male breast cancer. The magnitude of the ORs in the current study are within the range observed for female breast cancer in the Cape Cod study and studies of occupational exposures to solvents. Cumulative exposures to PCE, DCE and vinyl chloride were also observed to possibly accelerate the onset of male breast cancer in the exploratory analyses. The study did not find evidence suggesting associations between male breast cancer and exposures to benzene and a combined measure of the sum of all the contaminants in the drinking water at Camp Lejeune. Cumulative exposure to TCE was not associated with the risk of male breast cancer in the logistic regression analysis but was observed to accelerate the onset of male breast cancer in the exploratory analysis. However, the study was limited by small numbers of exposed cases and resulting wide CIs. For example, in the logistic regression analyses, the results for PCE and vinyl chloride were based on two cases with high exposure and the result for DCE was based on three cases with high exposure. Although the results of this study add to the scientific literature on the risk of male breast cancer incidence in a veteran population exposed to these chemicals in drinking water, additional research is needed.

## Additional files

Additional file 1:
**Data elements obtained from military personnel records for Camp Lejeune Male Breast Cancer Study.** (DOCX 14 kb) Additional file 2:
**Data variables requested from the Department of Veterans Affairs Central Cancer Registry (VACCR) and Patient Treatment Files (PTF).** (DOCX 12 kb) Additional file 3: Figure S8. Splines for PCE cumulative exposure (μg/L-year) and male breast cancer using RCS with 3 knots (25th, 50th, and 75th percentiles among those with exposure). **Figure S9.** Splines for TCE cumulative exposure (μg/L-year) and male breast cancer using RCS with 3 knots (10^th^, 50^th^, and 90^th^ percentiles among those with exposure). **Figure S10.** Splines for DCE cumulative exposure (μg/L-year) and male breast cancer using RCS with 3 knots (10^th^, 50^th^, and 90^th^ percentiles among those with exposure). **Figure S11.** Splines for vinyl chloride cumulative exposure (μg/L-year) and male breast cancer using RCS with 3 knots (20^th^, 50^th^, and 80^th^ percentiles among those with exposure). **Figure S12.** Splines for benzene cumulative exposure (μg/L-year) and male breast cancer using RCS with 3 knots (10^th^, 50^th^, and 90^th^ percentiles among those with exposure). **Figure S13.** Splines for TVOC cumulative exposure (μg/L-year) and male breast cancer using RCS with 3 knots (5^th^, 50^th^, and 95^th^ percentiles among those with exposure). **Figure S14.** Splines for PCE average exposure (ppb) and male breast cancer using RCS with 3 knots (25th, 50th and 75th percentiles among those with exposure). **Figure S15.** Splines for DCE average exposure (ppb) and male breast cancer using RCS with 3 knots (20th, 50th, and 80th percentiles among those with exposure). (DOCX 380 kb)

## Abbreviations

aOR: Adjusted odds ratio
ATSDR: Agency for Toxic Substances and Disease Registry
BOQ: Bachelor officers’ quarters
CDC: Centers for Disease Control and Prevention
CI: Confidence interval
DCE: t- 1,2-dichloroethylene
EMF: Electromagnetic fields
HP: Hadnot point
HB: Holcomb Boulevard
HR: Hazard ratio
ICD: International Classification of Diseases
MCL: Maximum contaminant level
*μ*g: Micrograms
MOS: Military occupational specialty
NHIA: North American Association of Central Cancer Registries Hispanic Identification Algorithm
NPRC: National Personnel Records Center
OR: Odds ratio
PCE: Tetrachloroethylene
PPB: Parts per billion
PTF: Patient treatment file
SE: Standard error
SEER: Surveillance, Epidemiology, and End Results
TT: Tarawa Terrace
TCE: Trichloroethylene
TVOC: Sum of the amount of all the contaminants
USMC: United States Marine Corps
VA: Department of Veteran’s Affairs
VACCR: Department of Veteran’s Affairs Central Cancer Registry
VOCs: Volatile organic compounds
WTP: Water treatment plant
