# Supplementary material for: Evaluation of contaminated drinking water and male breast cancer at Marine Corps Base Camp Lejeune, North Carolina: a case control study
Source: Environ Health. 2015 Sep 16;14:74. doi: 10.1186/s12940-015-0061-4 (PMC4571057; doi:10.1186/s12940-015-0061-4)
Supplement: Additional file 1: — Data elements obtained from military personnel records for Camp Lejeune Male Breast Cancer Study. (DOCX 14 kb) [file 12940_2015_61_MOESM1_ESM.docx]

**Additional File 1. Data elements obtained from military personnel records for Camp Lejeune Male Breast Cancer Study**

| **Category** | **Specific variables** |
| --- | --- |
| Personal identifying information | Name, social security number, Marine Corp service number, date of birth |
| Tour(s) of active and reserve duty | Start and end dates |
| Rank(s) | Date(s) of promotions and demotions |
| Military occupational specialty (MOS) | MOS codes and start dates for each occupational series |
| Station assignment at Camp Lejeune | Unit, arrival and departure dates |
| Service in Vietnam | Arrival and departure dates |
| Deployments (e.g. unit change, training, combat, etc.) for Marines at Camp Lejeune | Description. arrival and departure dates |
| Marital status for all Marines; dependent status for Marines at Camp Lejeune | Date of marriage(s)/divorce(s), date(s) of birth for child(ren), addresses for all dependents, dates of arrival and departure for each address |
