# Supplementary material for: Evaluation of contaminated drinking water and male breast cancer at Marine Corps Base Camp Lejeune, North Carolina: a case control study
Source: Environ Health. 2015 Sep 16;14:74. doi: 10.1186/s12940-015-0061-4 (PMC4571057; doi:10.1186/s12940-015-0061-4)
Supplement: Additional file 2: — Data variables requested from the Department of Veterans Affairs Central Cancer Registry (VACCR) and Patient Treatment Files (PTF). (DOCX 12 kb) [file 12940_2015_61_MOESM2_ESM.docx]

**Additional File 2. Data variables requested from the Department of Veterans Affairs Central Cancer Registry (VACCR) and Patient Treatment Files (PTF)**

| **VACCR** | **VA PTF** |
| --- | --- |
| Social Security Number | Social Security Number |
| Last Name | Last Name |
| Middle Name | Middle Name |
| First Name | First Name |
| Address at Diagnosis – State | Date of Birth |
| Race | Race |
| Ethnicity | Diagnosis codes (ICD 9) |
| NHIA* Derived Hispanic Origin | Date(s) of admittance for each diagnosis |
| Sex | Age at Admittance |
| Date of Birth |  |
| Age at Diagnosis |  |
| Date of Diagnosis |  |
| Weight at Diagnosis |  |
| Height at Diagnosis |  |
| Primary Site |  |
| Multiple Primary Site |  |
| Date of Multiple Tumors |  |
| Laterality |  |
| Histologic type/Behavior code |  |
| Grade |  |
| Diagnostic confirmation |  |
| SEER† summary stage – 2000 |  |
| SEER† summary stage – 1977 |  |
| Derived SEER† Summary Stage – 2000 |  |
| Sequence Number – Central |  |
| Diagnosis codes (ICD 9/10 codes) for co-morbidities |  |
| Dates of diagnosis for co-morbidities |  |
| Family History of Cancer |  |

* North American Association of Central Cancer Registries Hispanic Identification Algorithm

† Surveillance, Epidemiology, and End Results
