# Supplementary material for: Evaluation of contaminated drinking water and male breast cancer at Marine Corps Base Camp Lejeune, North Carolina: a case control study
Source: Environ Health. 2015 Sep 16;14:74. doi: 10.1186/s12940-015-0061-4 (PMC4571057; doi:10.1186/s12940-015-0061-4)

Figure 8. Splines for PCE cumulative exposure (µg/L-year) and male breast cancer using RCS with 3 knots (25th, 50th, and 75th percentiles among those with exposure)


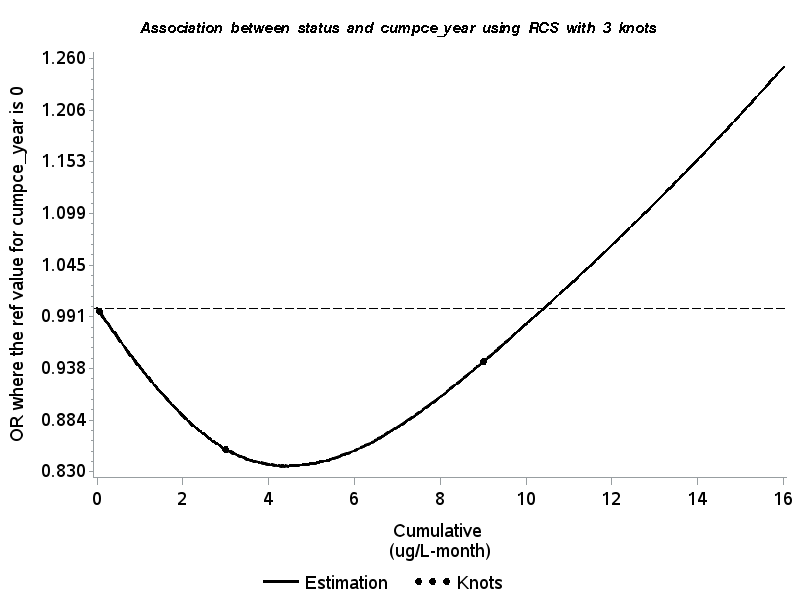


**^. . .^** ^Knots^


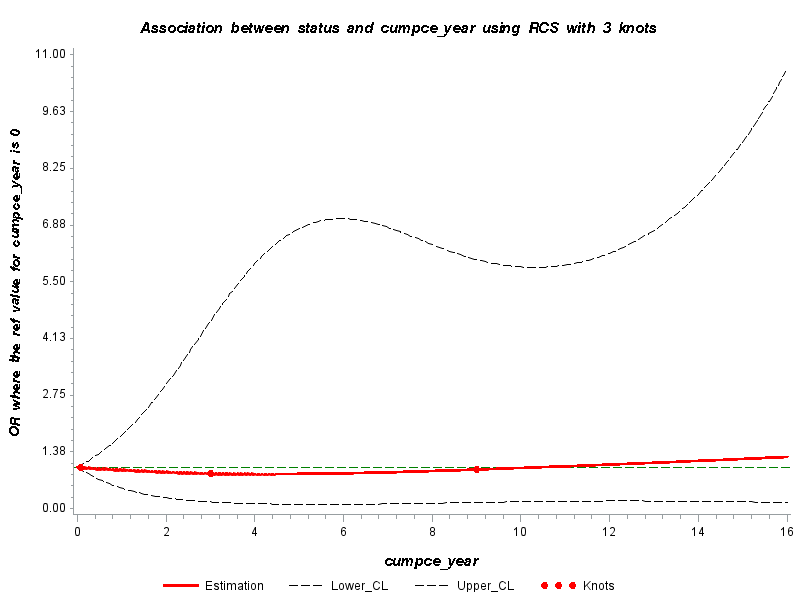


Figure 9. Splines for TCE cumulative exposure (µg/L-year) and male breast cancer using RCS with 3 knots (10^th^, 50^th^, and 90^th^ percentiles among those with exposure)


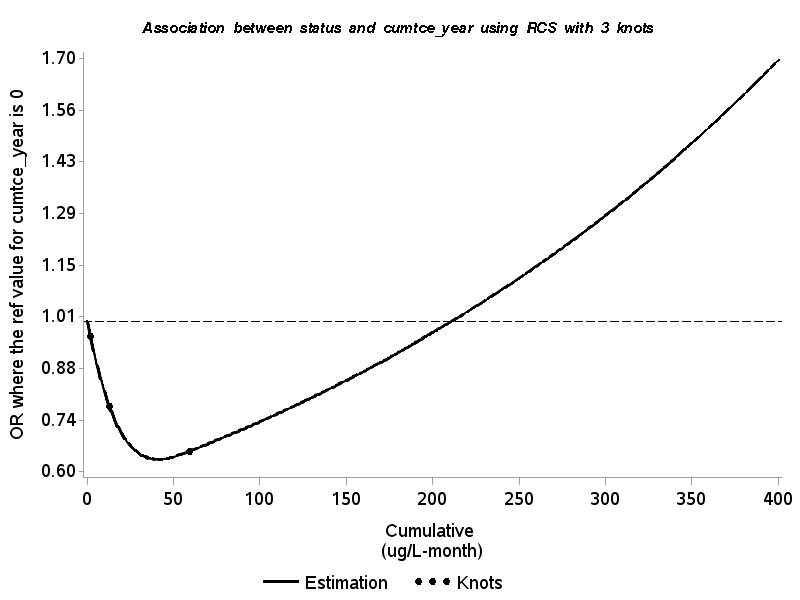


**^. . .^** ^Knots^


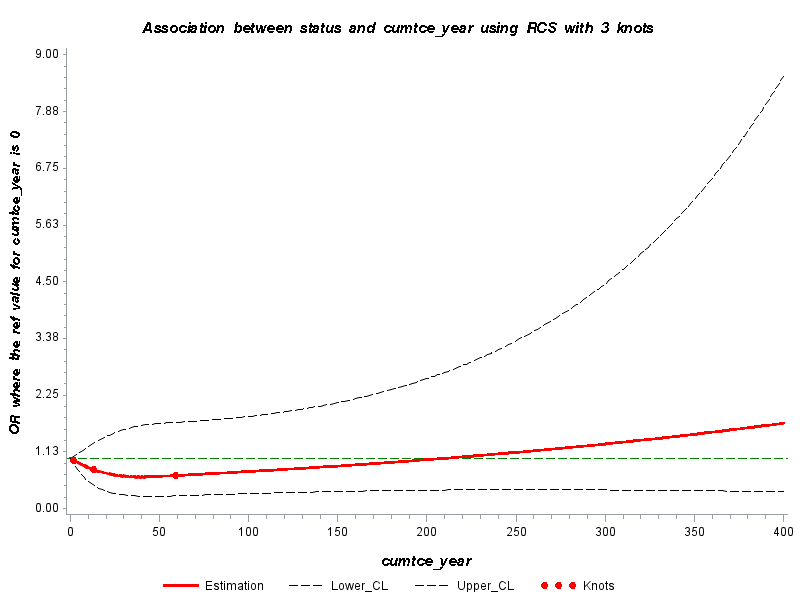


Figure 10. Splines for DCE cumulative exposure (µg/L-year) and male breast cancer using RCS with 3 knots (10^th^, 50^th^, and 90^th^ percentiles among those with exposure)


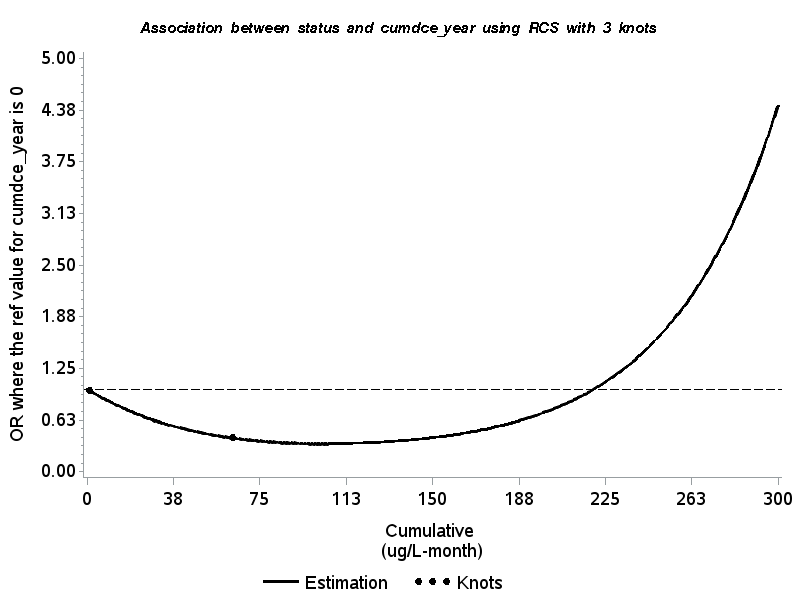


**^. . .^** ^Knots^


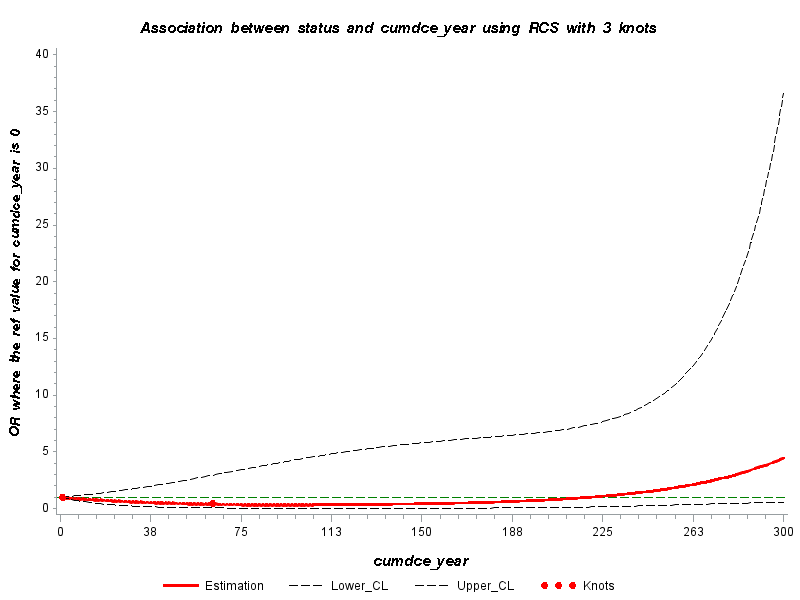


Figure 11. Splines for vinyl chloride cumulative exposure (µg/L-year) and male breast cancer using RCS with 3 knots (20^th^, 50^th^, and 80^th^ percentiles among those with exposure)


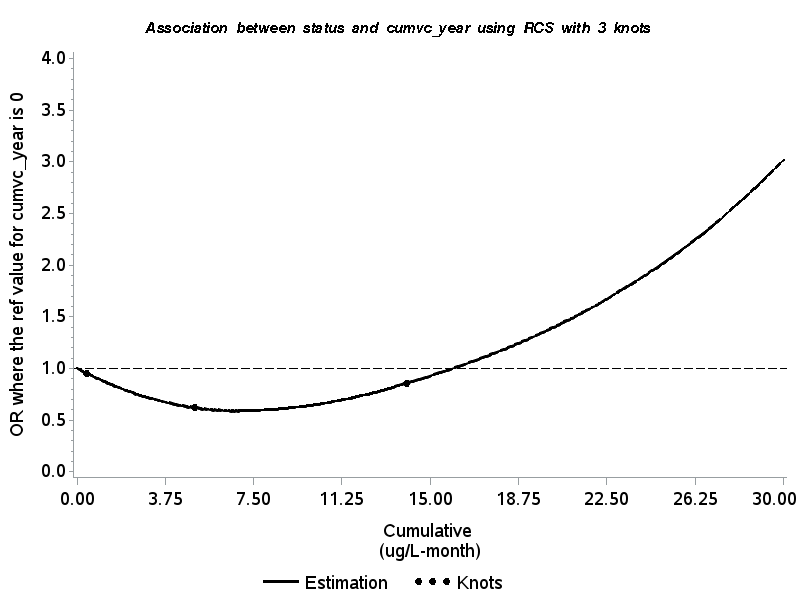


**^. . .^** ^Knots^


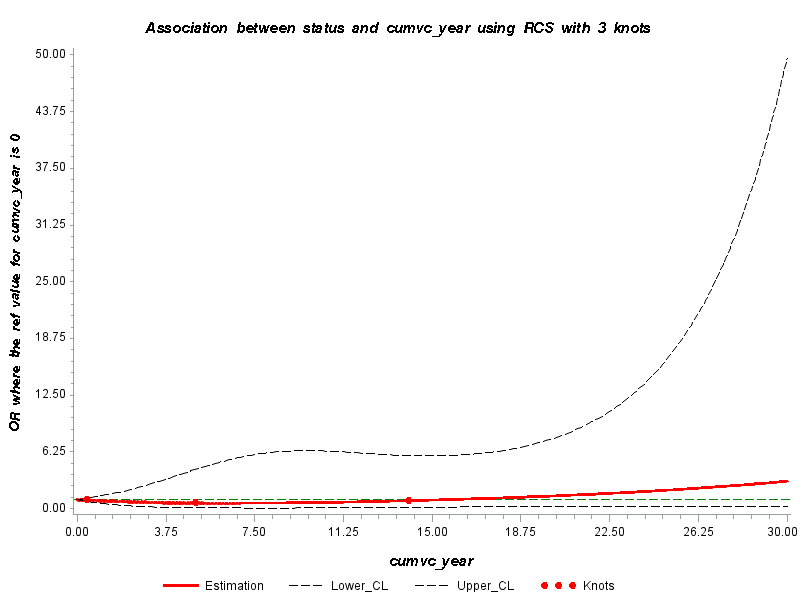


Figure 12. Splines for benzene cumulative exposure (µg/L-year) and male breast cancer using RCS with 3 knots (10^th^, 50^th^, and 90^th^ percentiles among those with exposure)
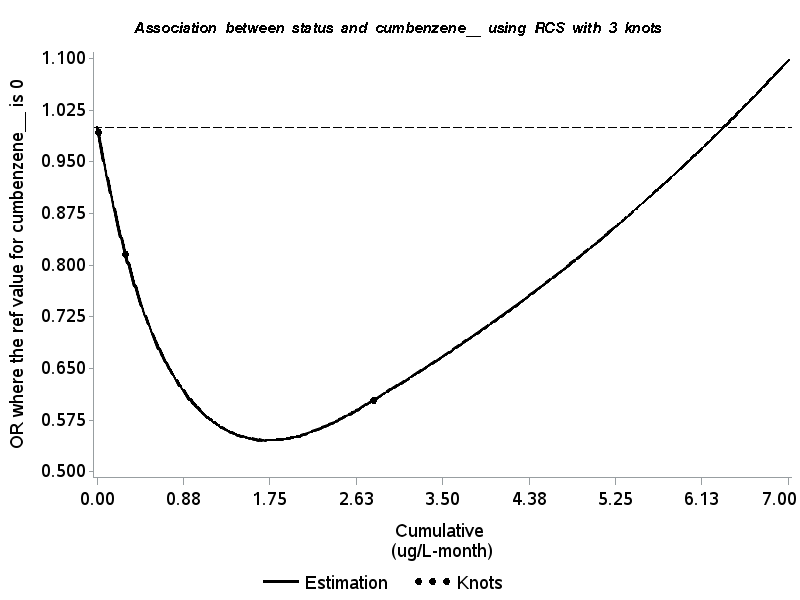


**^. . .^** ^Knots^


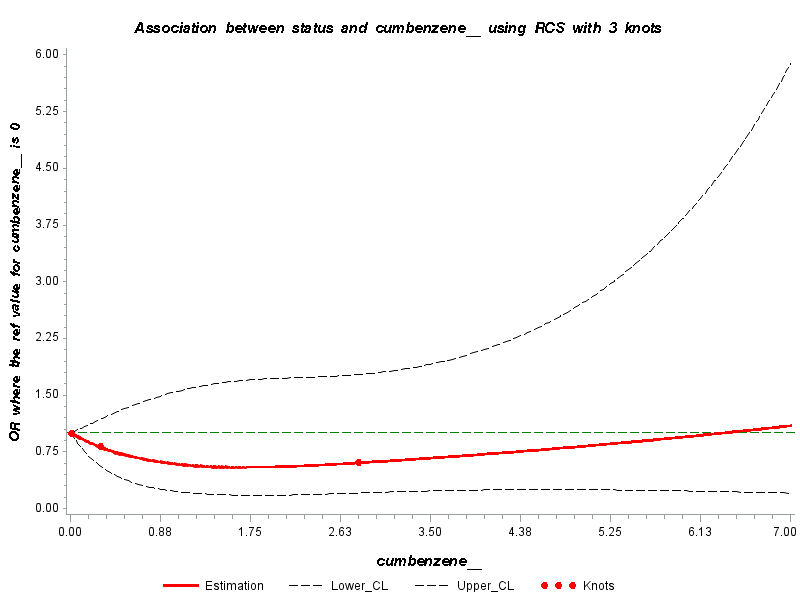


Figure 13. Splines for TVOC cumulative exposure (µg/L-year) and male breast cancer using RCS with 3 knots (5^th^, 50^th^, and 95^th^ percentiles among those with exposure)


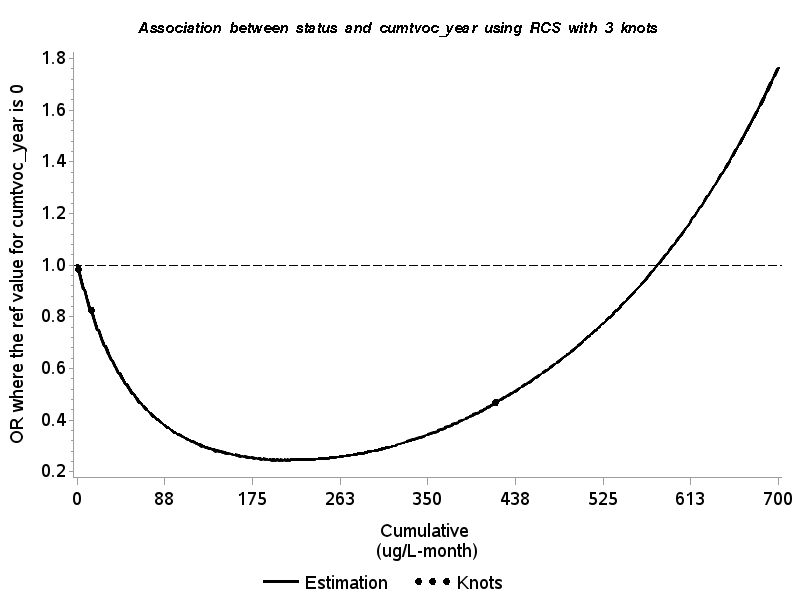


**^. . .^** ^Knots^

^
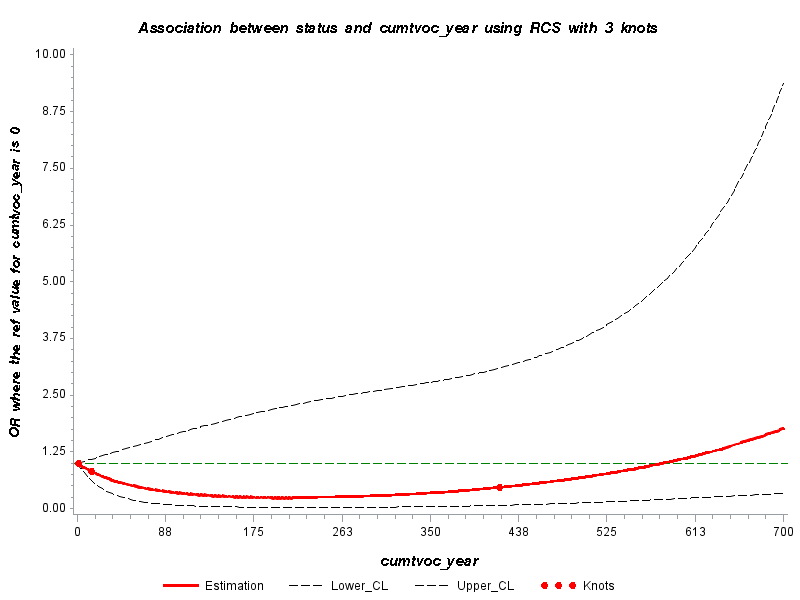
^

^Figure 14. Splines for PCE average exposure (ppb) and male breast cancer using RCS with 3 knots (25th, 50th and 75th percentiles among those with exposure)^

^
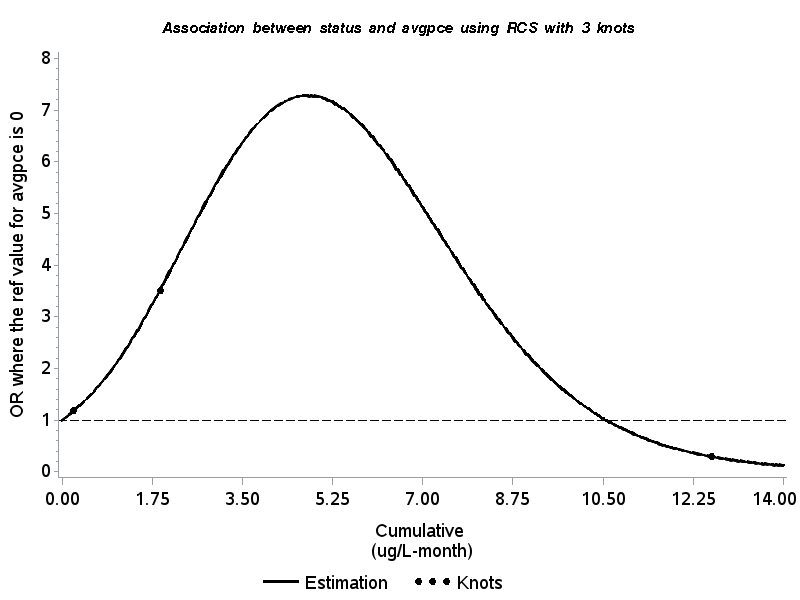
^

**^. . .^** ^Knots^

^
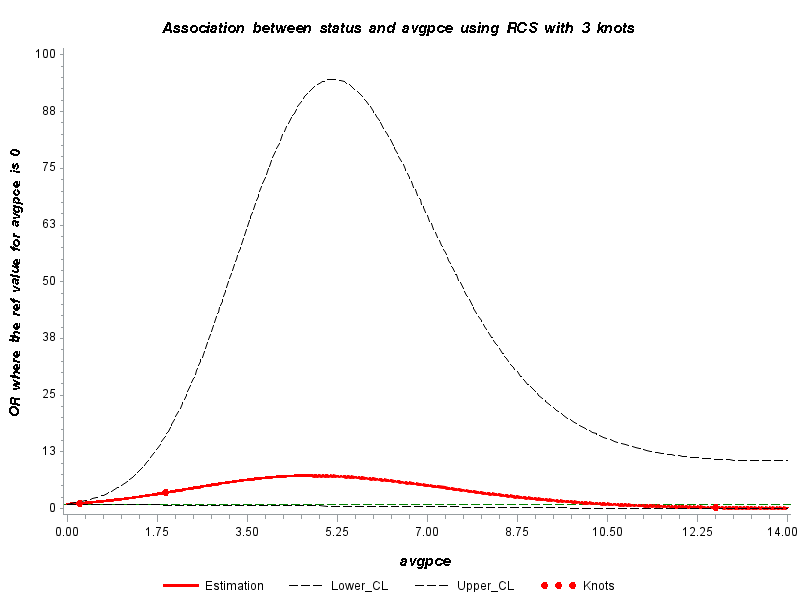
^

^Figure 15. Splines for DCE average exposure (ppb) and male breast cancer using RCS with 3 knots (20th, 50th, and 80th percentiles among those with exposure)^


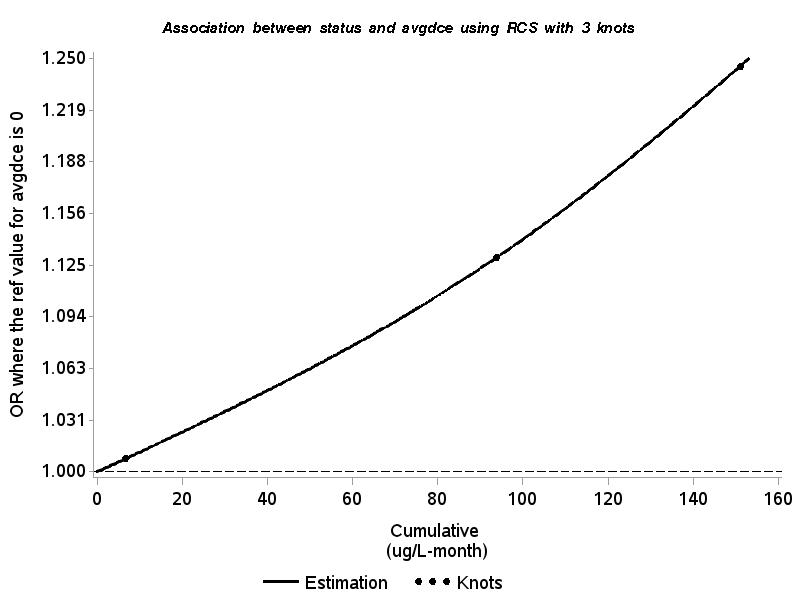


**^. . .^** ^Knots^


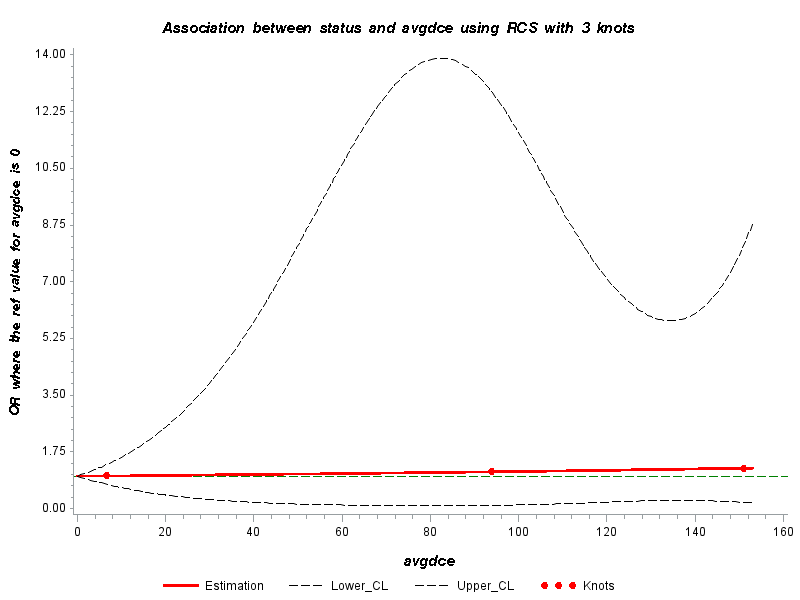

Supplement: Additional file 3: Figure S8. — Splines for PCE cumulative exposure (μg/L-year) and male breast cancer using RCS with 3 knots (25th, 50th, and 75th percentiles among those with exposure). Figure S9. Splines for TCE cumulative exposure (μg/L-year) and male breast cancer using RCS with 3 knots (10th, 50th, and 90th percentiles among those with exposure). Figure S10. Splines for DCE cumulative exposure (μg/L-year) and male breast cancer using RCS with 3 knots (10th, 50th, and 90th percentiles among those with exposure). Figure S11. Splines for vinyl chloride cumulative exposure (μg/L-year) and male breast cancer using RCS with 3 knots (20th, 50th, and 80th percentiles among those with exposure). Figure S12. Splines for benzene cumulative exposure (μg/L-year) and male breast cancer using RCS with 3 knots (10th, 50th, and 90th percentiles among those with exposure). Figure S13. Splines for TVOC cumulative exposure (μg/L-year) and male breast cancer using RCS with 3 knots (5th, 50th, and 95th percentiles among those with exposure). Figure S14. Splines for PCE average exposure (ppb) and male breast cancer using RCS with 3 knots (25th, 50th and 75th percentiles among those with exposure). Figure S15. Splines for DCE average exposure (ppb) and male breast cancer using RCS with 3 knots (20th, 50th, and 80th percentiles among those with exposure). (DOCX 380 kb) [file 12940_2015_61_MOESM3_ESM.docx]
